# In How Many Ways is the Approximate Number System Associated with Exact Calculation?

**DOI:** 10.1371/journal.pone.0111155

**Published:** 2014-11-19

**Authors:** Pedro Pinheiro-Chagas, Guilherme Wood, André Knops, Helga Krinzinger, Jan Lonnemann, Isabella Starling-Alves, Klaus Willmes, Vitor Geraldi Haase

**Affiliations:** 1 Cognitive Neuroimaging Unit, Institut National de la Santé et de la Recherche Médicale (INSERM) U992, Gif/Yvette, France; 2 NeuroSpin Center, Institute of BioImaging Commissariat à l'Energie Atomique (CEA), Gif/Yvette, France; 3 Developmental Neuropsychology Laboratory, Department of Psychology, Universidade Federal de Minas Gerais (UFMG), Belo Horizonte, Brazil; 4 Department of Neuropsychology, Institute of Psychology, Karl-Franzens University of Graz, Graz, Austria; 5 Faculty of Life Sciences, Humboldt Universität zu Berlin, Berlin, Germany; 6 Section Child Neuropsychology, Department of Child and Adolescent Psychiatry, University Hospital of the RWTH, Aachen, Germany; 7 German Institute for International Educational Research, Frankfurt, Germany; 8 Center for Individual Development and Adaptive Education of Children at Risk (IDeA), Frankfurt, Germany; 9 Section Neuropsychology, Department of Neurology, University Hospital of the RWTH, Aachen, Germany; 10 Programa de Pós-graduação em Neurociências, Universidade Federal de Minas Gerais (UFMG), Belo Horizonte, Brazil; 11 Programa de Pós-graduação em Saúde da Criança e do Adolescente, Faculdade de Medicina, Universidade Federal de Minas Gerais (UFMG), Belo Horizonte, Brazil; The University of Western Ontario, Canada

## Abstract

The approximate number system (ANS) has been consistently found to be associated with math achievement. However, little is known about the interactions between the different instantiations of the ANS and in how many ways they are related to exact calculation. In a cross-sectional design, we investigated the relationship between three measures of ANS acuity (non-symbolic comparison, non-symbolic estimation and non-symbolic addition), their cross-sectional trajectories and specific contributions to exact calculation. Children with mathematical difficulties (MD) and typically achieving (TA) controls attending the first six years of formal schooling participated in the study. The MD group exhibited impairments in multiple instantiations of the ANS compared to their TA peers. The ANS acuity measured by all three tasks positively correlated with age in TA children, while no correlation was found between non-symbolic comparison and age in the MD group. The measures of ANS acuity significantly correlated with each other, reflecting at least in part a common numerosity code. Crucially, we found that non-symbolic estimation partially and non-symbolic addition fully mediated the effects of non-symbolic comparison in exact calculation.

## Introduction

An extensive literature that comprises psychophysical [1], [2], electrophysiological [3], and neuroimaging data [4] has demonstrated that human infants and adults share an approximate number system (ANS), which is dedicated to representing large magnitudes in an analog fashion. Number representation within the ANS is very similar to the intuition that we have for space and time magnitudes [5] and can be well described by Weber-Fechner's law [3], [4], [6], [7]. Because the ANS is already present in newborns [8] and interacts with culturally derived symbolic representations during development [9], it is considered to be an important *start-up* tool for the acquisition of mathematical knowledge [10].

Converging evidence from correlational [11], cross-sectional [12], [13], longitudinal [14]–[18] and training studies [19] has provided robust support for the link between the ANS and arithmetics. Halberda et al. [11] showed that the Weber fraction calculated from a non-symbolic number comparison task in adolescents retroactively correlated with standardized math achievement scores from Kindergarten up to the sixth grade. A series of other studies replicated this finding using not only general standardized math achievement scores [18], [20]–[22] but also simple arithmetics operations [13], [23].

Importantly, a number of other studies failed to find an association between non-symbolic comparison and math achievement, but rather found significant associations between math achievement and the symbolic version of the task (see [24] for a review of the inconsistency of those findings).

However, two recent meta-analyses confirmed the existence of a robust association between non-symbolic comparison and math achievement from childhood to adulthood. Fazio, Bailey, Thompson and Siegler [25] analyzed 19 published studies and found that although non-symbolic processing is less strongly correlated with math achievement compared to symbolic processing, there is a robust and specific significant association between non-symbolic comparison and math achievement. Chen and Li [26] investigated 36 cross-sectional studies and found that the association between non-symbolic comparison and math achievement is moderate but statistically significant (r = 0.20, 95% CI = [0.14, 0.26]), even after controlling the effect of general cognitive abilities. Importantly, non-symbolic comparison was found to prospectively predict later math performance (r = 0.24, 95% CI = [0.11, 0.37]; 6 samples) and it is also retrospectively correlated to early math achievement (r = 0.17, 95% CI = [0.07, 0.26]; 5 samples). Based on the estimated effect sizes, the authors conducted power analyses and confirmed that many previous studies failed to find a significant association between non-symbolic comparison and math achievement because of insufficient statistical power due to small sample sizes.

Moreover, other measures of ANS acuity, such as number estimation, were also found to correlate with math achievement in children and adolescents [12], [16], [27].

Noticeably, longitudinal and training studies have provided evidence for a foundational role of the ANS on the development of math abilities. Using the non-symbolic number comparison task, Mazzoco et al. [18] showed that the ANS acuity measured prior to formal mathematical instruction was selectively predictive of arithmetics achievement in the first grade (see also Libertus et al. [17]). Similarly, Gilmore et al. [15] found that non-symbolic calculation abilities measured in a group of Kindergarten children were a robust predictor of later math achievement. Complementary, Park and Brannon [19] showed that training adults in a non-symbolic addition and subtraction task specifically improves exact addition and subtraction. Interestingly, the ANS acuity was also found to improve with mathematical education. Piazza, Pica, Izard, Spelke, and Dehaene [28] investigated a group of Mundurukus, an indigenous population in Brazil that does not have a system for representing exact numbers [29], and found that the ANS acuity, as quantified by a non-symbolic number comparison task, was modulated by the level of formal instruction at the standard Brazilian school system. This result provides support for a bidirectional association between the most basic forms of number processing and math abilities. Importantly, an analogous bidirectionality has long been found in the reading domain, such as the fact that phonological abilities serve as the base for reading competence and are improved by literacy [30], [31].

Finally, group studies demonstrated that children with developmental dyscalculia (DD), a learning disability specific to calculation, have an impaired ANS compared to their typically achieving (TA) peers. Piazza et al. [13] showed that the ANS acuity in children with DD at 10 years old, as quantified by the internal Weber fraction, was equivalent to the acuity observed in TA Kindergarten children. Similar results were obtained by Mazzoco et al. [12], who showed not only that adolescents with DD present higher internal Weber fractions than their TA peers but also that they have an impairment in estimating numerical magnitudes.

Other studies that investigated younger children with DD found only an impairment in the symbolic version of the number comparison task (see review by Noël & Rousselle [32]), which casts doubt on the assumption of a critical role of the ANS in the acquisition of exact number representations. Chu, van Marle, and Geary [33] found that the ANS acuity significantly predicted the risk for DD in children, but measures of symbolic number knowledge were more robust predictors. However, those studies used only one measure to assess the ANS acuity: the non-symbolic number comparison task. Based on Gilmore et al. [15] it could be the case that different forms of approximate manipulation of numerical information, such as calculation, could be additional important predictors of risk for DD.

Although much progress has been achieved in the establishment of an association between the ANS and arithmetics, it remains largely elusive in how many ways the ANS interacts with exact calculation and through which cognitive mechanisms this association could be grounded. The ANS allows for comparing two different magnitudes, to approximately grasp how many objects are present in a scene and to manipulate quantities using simple operations such as addition and subtraction [34]. In this sense, different tasks have been used to measure the ANS acuity, such as comparison [11]–[13], [35], estimation [12], [23], [27], [36] and approximate calculation [15], [37], [38]. Importantly, very little attention was given to the fact that these measures are tapping different instantiations of the ANS. Comparison, estimation and approximate calculation, although possibly operating at the same level of representation (the ANS), involve very different computational processes and consequently could have specific contributions to the development of exact number representations and mathematics. Indeed, Mazzocco et al. [12] found that non-symbolic comparison and estimation accounted for unique proportions of the variance when predicting math achievement.

Moreover, given the complexity of arithmetics, the link between basic number processing (e.g., magnitude comparison) and exact calculation is possibly not direct and might involve the recruitment of other cognitive processes. Indeed, the study by Lyons and Beilock [39] found that the ability to identify the order of a series of digits fully mediated the association between the ANS acuity (as measured with the non-symbolic number comparison task) and exact calculation in adults. Importantly, van Marle, Chu, Li and Geary [40] provided a conceptual replication of the study of Lyons and Beilock [39] in children, and proposed that the ANS acuity facilitated the early acquisition of symbolic number knowledge and was indirectly associated with math achievement through this knowledge. In line with these findings, it might also be the case that there is a type of hierarchical association between different instantiations of the ANS, from the most elementary abilities to more complex operations and manipulations of magnitude information. That is, non-symbolic estimation and calculation could be intermediate steps between simple number discrimination and exact calculation.

Surprisingly, to date there is only one study in adults and no study with children that directly compared different measures of the ANS. Gilmore, Attridge, and Inglis [41] measured the ANS with non-symbolic versions of the number comparison and approximate addition tasks and found null correlations between the internal Weber fractions calculated from each task, placing in doubt the assumption of a single underlying ANS. However, this result is very puzzling and deserves further examination, because both tasks used non-symbolic magnitudes and, even though different cognitive mechanisms might be recruited during performance, both tasks should at least partially activate the representation of numbers and its underlying brain circuitry. Indeed, using a conjunction analysis, Park, Park and Polk [42] recently showed that non-symbolic comparison and non-symbolic addition activated common brain circuitries in the right parietal cortex.

Therefore, a more comprehensive investigation of the association between different instantiations of the ANS (comparison, estimation and calculation), their cross-sectional trajectories in children with typical and atypical math abilities and how they interact with exact calculation is needed.

### The present study

Measures that are related to the ANS acuity appear to be normally distributed in the population [11] and are systematically associated with arithmetics achievement. In this sense, the present study first addressed the hypothesis that children with *math difficulties* (MD) who were selected according to a relatively liberal criterion (below the 25^th^ percentile on a standardized math achievement test [43]) would present with lower ANS acuity compared to their TA peers. To this end, we calculated specific psychophysical parameters for each of three different tasks as indices of ANS acuity. The internal Weber fraction (*w*) [1] was calculated for the non-symbolic number comparison task, and the coefficient of variation (*cv*) was calculated for the non-symbolic estimation and non-symbolic addition tasks. The *cv* is a normalized measure of dispersion of a probability distribution and it is defined as the ratio of the standard deviation to the mean. Therefore, like the *w*, the higher the *cv*, the lower the precision. Based on the previous results obtained by Mazzocco et al. [12] and Piazza et al. [13], we expected TA children to have higher ANS acuity (lower values in the psychophysical parameters) compared to children with MD. Second, as noted by Noël and Rousselle [32], one should expect to find differences between the TA and MD groups in the cross-sectional trajectories of the ANS.

More specifically, group differences in ANS acuity, at least as measured by non-symbolic number comparison, should have a trend to increase across development. We finally tested the degree of association between the measures of ANS acuity and exact calculation. Because the psychophysical parameters extracted from the tasks that measure the ANS acuity are at least partially related to the degree of noise in the representation of numerosity, they should be positively correlated to one another. Moreover, based on previous studies [11], [18], it is expected that the ANS acuity will have a specific impact on exact calculation, even after controlling for the effects of general developmental factors and other abilities that are related to mathematics, such as language. Crucially, based on the results by Lyons and Beilock [39], who showed that number ordering fully mediated the effect of non-symbolic comparison in exact calculation, we further investigated the relationship between the ANS acuity and calculation using mediation models. Six mediation models were estimated with all possible permutations between measures of ANS acuity as predictors or mediators and exact calculation as the outcome.

## Materials and Methods

### Participants

This study was approved by the ethics review board of the Federal University of Minas Gerais, Brazil (COEP–UFMG). Informed consent was obtained in written form from the parents and orally from the children. Children from first to sixth grade were recruited from public and private schools in Brazil and were assigned to different groups according to their performance in the Arithmetics and word Spelling subtests of the Brazilian School Achievement Test (Teste de Desempenho Escolar, TDE [44]). The typical achievement group (TA, n = 172) was composed of children who performed above the 25^th^ percentile in both the Arithmetics and Spelling subtests of TDE. The mathematical difficulties group (MD, n = 45) performed below the 25^th^ percentile in the Arithmetics and above that in the Spelling subtest of the TDE.

There were no statistically significant differences in age and sex between groups. All of the children had normal intelligence, as measured by Raven's Colored Progressive Matrices (IQ scores above 85).

Children were assessed using an exact calculation task comprising addition, subtraction and multiplication, a simple reaction time task and three tasks that measured the ANS acuity: non-symbolic comparison, non-symbolic estimation and non-symbolic addition (see the detailed description of the tasks below).

A subgroup of 10 children from the TA and 5 from the MD group were excluded from further analyses, because either they had a poor fit (*R^2^*) for estimation of the *w* on the non-symbolic comparison task (*R^2^*<0.2), and/or they showed a *w* that exceeded the limit of discriminability of the comparison task (*w*>0.6). The final sample was composed of 162 TA children and 40 children with MD. The subject details are presented in Table 1 (for the descriptive data of the individual assessment samples by grade, see Table S1 in the Supporting Information).

**Table 1:** Descriptive data of the individual assessment sample.

Categorical Variables	TA (n = 162)	MD (n = 40)	*χ^2^*	*df*	*p-value*
Sex (% female)	59.26	52.50	0.601	1	0.274
School type (% public)	86.42	87.50	0.032	1	0.547

Continuous Variables	Mean	SD	Mean	SD	*t*	*df*	*p-value*	*d*
Age (months)	121.562	13.599	118.325	16.513	1.290	200	0.199	0.228
Raven (IQ score)	110.612	10.545	103.619	9.659	3.817	200	<0.001	0.674
TDE Arithmetics	108.924	11.406	85.784	5.073	12.510	200	<0.001	2.209
TDE Spelling	110.078	8.130	101.220	8.929	6.050	200	<0.001	1.068

TA: typically achieving; MD: mathematical difficulties. Both TDE Arithmetics and TDE Spelling scores are in a standardized form with mean = 100 and SD = 15; d = Cohen's d.

### Tasks

#### The Brazilian School Achievement Test

The TDE [44] is the most widely used standardized test of school achievement that has norms for the Brazilian population (see also Oliveira-Ferreira et al. [45]). We used the Arithmetics and Spelling subtests, which can be applied in groups. Norms are provided for school-aged children between first and sixth grade. The Arithmetics subtest is composed of three simple verbally presented word problems (e.g., “If you had three candies and received four, how many candies do you have now?”) and 35 written arithmetic calculations of increasing complexity (e.g., very easy: “4–1”; easy: “1230+150+1620”; intermediate: “823 * 96”; hard: “3/4+2/8”). The Spelling subtest constitutes a dictation of 34 words that have increasing syllabic complexity (e.g., “toca”; “balanço”; “cristalização”). The reliability coefficients (*Cronbach*'*s α*) of the TDE subtests are 0.89 or higher. The children were instructed to work on the problems to the best of their capacity but without time limit.

#### Raven's Coloured Progressive Matrices

General intelligence was assessed with the Raven's Coloured Progressive Matrices, according to Brazilian norms [46].

#### Exact Calculation

The task was divided in two sets of items: symbolic and written verbal calculations. The symbolic calculation set was composed of additions (27 items), subtractions (27 items) and multiplications (28 items). Problems that were printed on separate sheets of paper. Children were instructed to answer as fast and as accurately as possible. Arithmetic operations were balanced at two levels of complexity and were presented to children in separate blocks: one block was composed of simple arithmetic table facts and the other block had more complex facts. Simple addition items had results below 10 (e.g., 3+5), while complex addition results ranged between 11 and 17 (e.g., 9+5). Tie problems (e.g., 4+4) were not considered for addition. Simple subtraction was composed of problems in which the operands were below 10 (e.g., 9–6), while in complex subtractions, the first operand ranged from 11 to 17 (e.g., 16–9). No negative results were included in the subtraction problems. Simple multiplication constituted operations that had results below 25 or that had the number 5 as one of the operands (e.g., 2 * 7, 5 * 6), whereas for the complex multiplication, the result of the operands ranged from 24 to 72 (e.g., 6 * 8). Tie problems were not used for multiplication. The time limit per block was set to 1 minute. The written verbal calculation set was composed of four additions and eight subtractions with single-digit operands (e.g. “Isabella has 9 cents. She gives 3 to Pedro. How many cents does Isabella have now?”). Problems were presented to children on a sheet of paper and read aloud by the examiner to avoid reading proficiency bias. The child had to solve the problems mentally and write down the answer in Arabic format as fast and as accurately as possible. The time limit per problem was 1 minute. The total score was calculated as a simple sum of all correct answers combining both symbolic and written verbal items (max score = 94). The task was highly reliable (all *Cronbach*'*s α*>0.90) [45], [47].

#### Simple Reaction Time

The computerized Reaction Time (RT) task was a simple task in which a picture of a wolf (height = 9.31 cm; length = 11.59 cm) was displayed in the center of a black screen for a maximum time of 3,000 ms [47]. Upon appearance of the wolf on screen, children were instructed to press the spacebar on the keyboard at the moment they saw the wolf, as fast as possible. Trials terminated with the first key press. The task had 30 trials, with an inter-trial interval of 2,000 ms, 3,500 ms, 5,000 ms, 6,500 ms or 8,000 ms. This task was used to control for possible differences in basic processing speed that were not related to numerical tasks.

#### Non-symbolic Comparison

**Figure 1:**
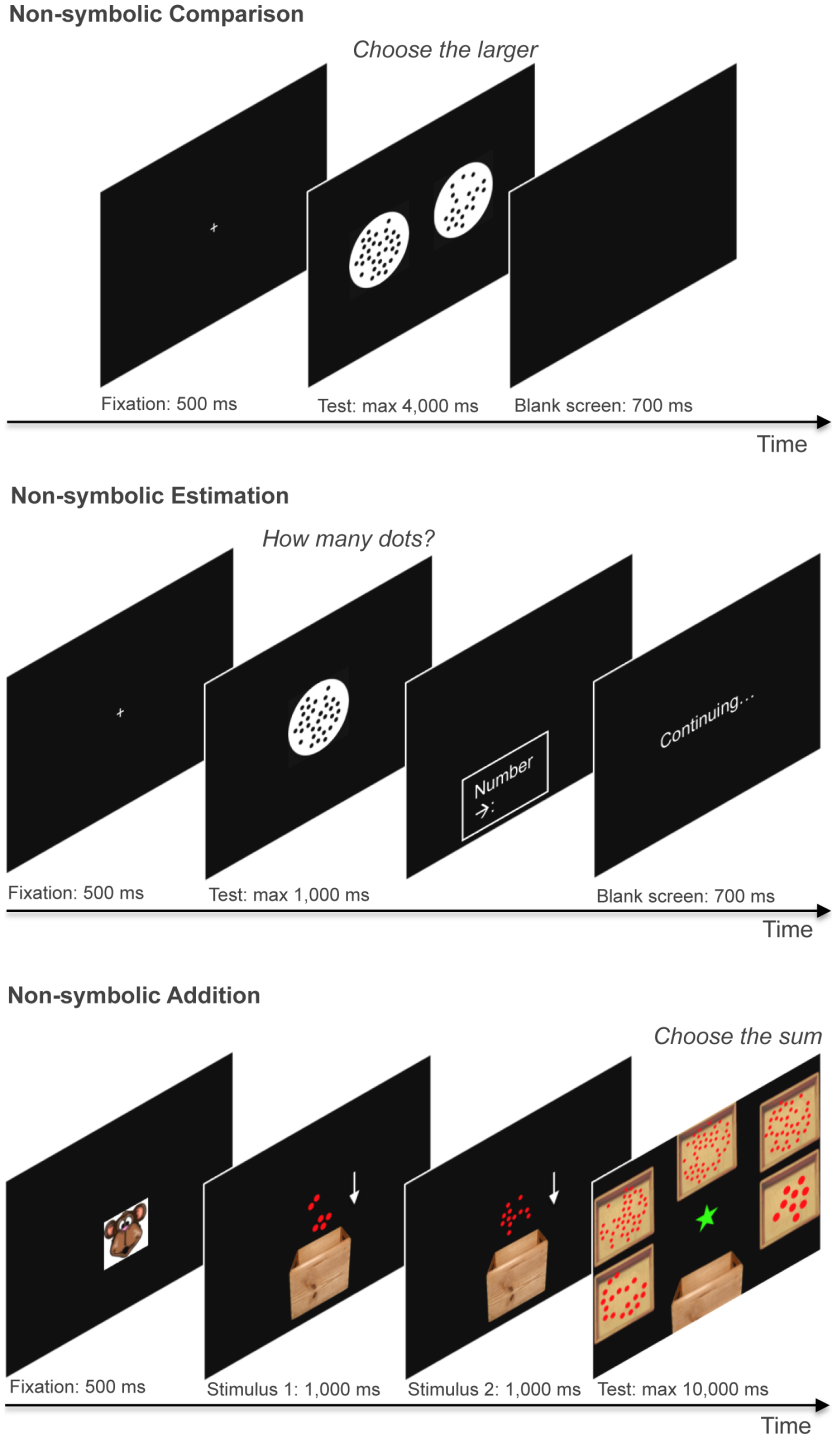
Psychophysical tasks used to measure ANS acuity, with non-symbolic comparison, non-symbolic estimation and non-symbolic addition. The white arrows are used in the bottom picture to illustrate the movement of the dots into the box.

In the non-symbolic comparison task, participants were instructed to compare two sets of black dots, which were simultaneously presented in two white circles on the left and on the right side of the screen, and they were instructed to choose the larger numerosity by pressing a key congruent to its side (left or right) (see Figure 1) [45], [47], [48]. Black dots were presented on a white circle against a black background. On each trial, one of the two white circles contained 32 dots (reference numerosity), and the other contained 20, 23, 26, 29, 35, 38, 41, or 44 dots. Each numerosity was presented eight times, and every presentation was arranged in a different configuration. The task comprised 64 testing trials. The maximum stimulus presentation time was 4,000 ms, and the intertrial interval was 700 ms. Between trials, a fixation point appeared on the screen for 500 ms; the fixation point was a cross printed in white and that had 3 cm for each line. To prevent the use of non-numerical cues, the sets of dots which represent the non-symbolic numerosities were designed and generated using a MATLAB script [49] such that on half of the trials, dot size remained constant, and total dot area covaried positively with the numerosity; on the other half of the trials, total dot area was held constant and dot size covaried negatively with numerosity. The data were trimmed for each child to exclude responses of ±3 SD from the individual mean RT. The *w* was calculated for each child as a measure of ANS acuity, based on the Log-Gaussian model of the number representation [1], with the methods described by Piazza et al. [4].

#### Non-symbolic Estimation

In the non-symbolic estimation task, participants were asked to estimate, with a verbal response, the quantity of dots shown on the computer screen [48] (see Figure 1). Black dots were presented on a white circle against a black background. The numerosities were 10, 16, 24, 32, 48, 56 or 64 dots. Each numerosity was presented 5 times, every time in a different configuration such that the same numerosity never appeared in consecutive trials. The task comprised 35 testing trials. To avoid counting, the maximum stimulus presentation time was set to 1000 ms. As soon as the child responded, the examiner, who was seated next to the child, pressed the spacebar on the keyboard and typed the child's answer. Between individual trials, a fixation point appeared on the screen, which was a cross printed in white, with 3 cm for each line. To prevent the use of non-numerical cues, the sets of dots were generated using MATLAB, in such a way that dot size changed but total dot area in a given set was always fixed across the stimuli. Thus, the total occupied area could not serve as a cue for distinguishing between the different numerosities. As a result of this manipulation, the average item size covaried inversely with numerosity. To avoid memorization effects due to the repetition of a specific stimulus, on each trial, the stimuli were randomly chosen from a set of 10 precomputed images with the given numerosity. The data were trimmed for each subject, to exclude the responses ±3 SD from the mean chosen value across all of the trials. As a measure of ANS acuity, we calculated the mean *cv* of the responses for each child.

#### Non-symbolic Addition

The non-symbolic addition task was based on Knops, Viarouge, and Dehaene [50] (see Figure 1). Participants were instructed to solve approximate addition problems with operands presented in a non-symbolic notation (dots patterns). To adapt the paradigm for the use of children, the addition task was embedded in a small history of a monkey having a box of balls. Hence, a trial started with the presentation of the monkey's face, which was followed by the appearance of a brown box against a black background and the first set of dots that moved into the box. Next, another set of dots moved into the same box. Afterward, the box disappeared from the screen and was replaced by the top-view of five boxes that contained different numerosities. The boxes were arranged in a circular manner around the middle of the screen. The children were to choose which numerosity was the closest to the correct outcome by clicking with the left mouse button on the respective box. The task comprised 2 learning trials and 32 testing trials. In the training trials, the boxes were framed after each response. In a case in which the response was correct, the frame was green, which indicated that the child had chosen the box with the correct number of balls. If the response was incorrect, then the frame was red, and the children were instructed to choose another box. This procedure was repeated until the child had chosen the correct box. Before starting the testing, the children were asked if they had understood the task, and if not, the training was repeated until they confirmed that they understood the task. In the testing period, the childrens' choices were indicated by a neutral blue frame around the chosen box, regardless of whether the response was correct or not. All of the addition problems added up to four possible results (i.e., 10, 16, 26 and 40), which combined ten different operands (4, 5, 6, 8, 10, 13, 14, 18, 20 or 26). To prevent the subjects from memorizing the problems, the operands were randomly “jittered" by adding a random value r, with r ∈ J and J = {−1,0,1}. For each correct response, 7 response alternatives were generated as round (c x 2.5^i/3^), where *c* is the correct result and *i* ranges from −3 to +3. To discourage the use of non-arithmetic strategies, such as “Always choose a response alternative in the middle of the presented range”, only five of the seven possible results were presented in a trial, such that, in half of the trials, the presented results were the upper five (high range), and thus, the correct response was the second largest numerosity. In the other half of the trials, the lower five results were shown (low range), and the correct response was the fourth largest numerosity. To prevent the use of non-numerical cues, the sets of dots were generated using MATLAB, in such a way that dot size covaried inversely with numerosity. To avoid memorization effects due to the repetition of a specific stimulus, on each trial, the stimuli were randomly chosen from a set of 10 precomputed images with the given numerosity. The data were trimmed for each subject, to exclude the responses ±3 SD from the mean chosen value across all of the trials. As a measure of ANS acuity, we calculated the mean *cv* of the four different results.

### Analyses

Initially, the TA and MD groups were compared with regard to exact calculation and the three measures of ANS acuity. Next, the cross-sectional trajectories of the ANS were investigated by calculating the slopes of the regressions between ANS acuity and age for each group separately. Finally, the association between the measures within the ANS and between the ANS acuity and the exact calculation was investigated in three steps. First, cross-correlations of the measures of ANS acuity and exact calculation were determined. Second, to estimate the specific contributions of ANS acuity measures to explain exact calculation, multiple regression models were conducted with exact calculation as the dependent variable and the three measures of ANS acuity as the predictor variables, regressing out the effects of age, schooling, general intelligence and spelling abilities. Finally, to investigate more deeply the possible mediation effects between the ANS instantiations and exact calculation, six mediation models were estimated with all of the possible permutations between the measures of ANS acuity as predictors or mediators and exact calculation as the outcome. All of the statistical analyses were performed using R statistical software [51]. Raw data is available in the Supporting Information (Data S1).

## Results

First, we verified whether the children's performances in the measures of ANS acuity followed Weber's law. In the non-symbolic comparison task, we calculated the *R^2^* of the fitting procedure to calculate the *w* for each child. In both the TA and MD groups, the *R^2^* values were high (TA: mean = 0.883, SD = 0.082; MD: mean = 0.849, SD = 0.108), which indicates that the children's performances were well described by the *Log-Gaussian* model of number representation [1]. For the non-symbolic estimation task, we calculated the coefficients of the regression between the correct outcomes and the mean *cv* per child in each presented numerosity. In both the TA and MD groups, the *β* coefficients were small (TA: mean = 0.145, SD = 0.513; MD: mean = 0.072, SD = 0.449), which indicates that children's responses have scalar variability. Nevertheless, the mean slope was significantly different from 0 in TA, but not in the MD group (TA: *t*(161) = 4.105, *p*<0.001; MD: *t*(39) = 0.883, *p* = 0.383). Similar *β* coefficients were obtained in the non-symbolic addition task, (TA: mean = 0.094, SD = 0.531; MD: mean = 0.056, SD = 0.581). In this case, the mean slope was not significantly different from zero in both groups (TA: *t*(161) = 0.122, *p* = 0.223; MD: *t*(39) = 1.119, *p* = 0.270). Taken together, these results demonstrate that the performance in all of the tasks that measure the ANS acuity from both the TA and MD groups can be well described by Weber's law. Group differences are presented in the next section.

### Differences between the TA and MD groups in ANS acuity

Although all of the children with MD had normal intelligence (IQ>85) and normal spelling achievement (above the 25^th^ percentile), they scored significantly lower in these measures when compared to their TA peers (Table 1; see Table S1 in the Supporting Information for the descriptive data separated by grade). For this reason, intelligence and spelling were included as covariates for group comparisons in exact calculation and ANS acuity (see Table 2 for statistics). As expected, the TA group showed better performance in exact calculation when compared to the children with MD. No group difference was found in the simple reaction time task. More importantly, the TA group presented higher ANS acuity, with significant lower *w* the non-symbolic comparison task. Moreover, TA children had lower *cv* in the non-symbolic estimation task; however, this difference was only marginally significant. Finally, a significantly lower *cv* was found in the non-symbolic addition task for the TA compared to the MD group.

**Table 2:** ANCOVAs comparing the TA and MD groups in mathematics and in the measures of ANS acuity controlling for intelligence and spelling.

	TA (n = 162)		MD (n = 40)					
Measures	Mean	SD	Mean	SD	*F*	*df*	*p-value*	*η^2^*
Exact Calculation (acc)	68.624	19.342	42.048	21.380	33.894	1;198	<0.001	0.146
Simple RT (ms)	416.876	84.201	444.911	102.362	2.363	1;198	0.126	0.012
Nsymb Comparison (w)	0.245	0.085	0.298	0.079	7.560	1;198	<0.01	0.037
Nsymb Estimation (cv)	0.278	0.086	0.312	0.111	3.273	1;198	0.072	0.016
Nsymb Addition (cv)	0.297	0.076	0.351	0.096	7.855	1;198	<0.01	0.038

TA  =  typically achieving; MD  =  mathematical difficulties; w: internal Weber fraction; cv: coefficient of variation; acc  =  accuracy in % correct.

### Cross-sectional trajectories of ANS acuity

**Figure 2:**
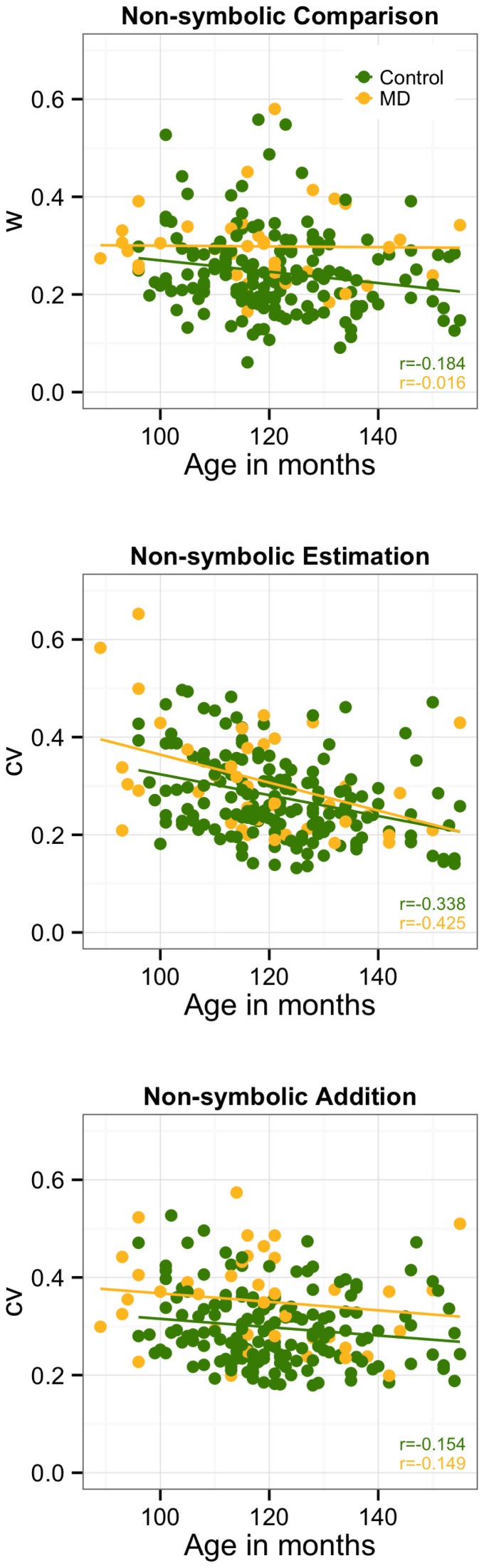
Cross-sectional trajectories of the measures of ANS acuity for the TA and MD groups.

Cross-sectional trajectories of the different measures of ANS acuity were investigated separately for the two groups (see Figure 2). The *w* was found to decrease monotonically with age in the TA children (*β* = −0.184, *p* = 0.015), but it remained stable in the children with MD (*β* = −0.016, *p* = 0.897). This result suggests that difference between the MD and TA groups in ANS acuity measured by the *w* increases during development. Second, the *cv* from non-symbolic estimation was found to monotonically decrease with age more or less to the same extent in both the TA and MD groups (TA: *β* = −0.338; *p*<0.001; MD: *β* = −0.425; *p* = 0.006). Finally, the *cv* from non-symbolic addition was also found to decrease with age by the same extent in both groups, but was only marginally significant in the TA group and was non-significant for the MD group (TA: *β* = −0.154, *p*<0.050; MD: *β* = −0.149, *p* = 0.375).

To confirm the results of the cross-sectional trajectory of the number comparison task, given the possible lack of statistical power in the MD group to detect significant coefficients, we ran a bootstrap analysis with the regression coefficients of the three measures of the ANS and age. First, we generated 10,000 samples with N = 40 (N of the MD group), allowing repetitive cases for each group separately. Next, we calculated for each sample one *β* coefficient for each of the regressions: age and non-symbolic comparison, age and non-symbolic estimation, and age and non-symbolic addition. Afterward, we calculated the percentage of positive coefficients in each group, which we use as a likelihood index for the true direction of the association in the population. In the non-symbolic comparison task, the coefficients of the TA group were found to be negative in 91.27% of the generated samples. This finding was not the case in the MD group, in which only 53.67% of the samples showed negative coefficients. For the other measures of ANS acuity, TA and MD showed similar patterns (non-symbolic estimation: TA = 97.30%, MD = 99.30%; non-symbolic addition: TA: 80.98%, MD: 82∶61%). Considering a confidence interval of 90%, both groups showed developmental changes in non-symbolic estimation, but the results were less robust in non-symbolic addition. Crucially, TA showed significant improvement in non-symbolic comparison in contrast to their MD peers, who definitively did not show any sign of improvement in this task during development.

### Relationship between the measures of ANS acuity and exact calculation

As expected, all of the three measures of ANS acuity showed significant positive correlations among themselves, even after controlling for the effects of age, spelling and intelligence, which indicates that they share a common construct. Importantly, all three measures of ANS acuity also correlated with exact calculation (Table 3).

**Table 3:** Partial correlations between measures of ANS acuity and exact calculation, controlling for age, intelligence and spelling.

	TA and MD group (n = 202)		
Measures	*Exact Calculation*	*Nsymb Estimation (cv)*	*Nsymb Addition (cv)*
***Nsymb Comparison (w)***	−0.212*	0.253*	0.346**
***Nsymb Estimation (cv)***	−0.237**	-	0.249**
***Nsymb Addition (cv)***	−0.233**	-	-

TA  =  typically achieving; MD  =  mathematical difficulties; w: internal Weber fraction; cv: coefficient of variation. *Correlation is significant at the 0.05 level; **Correlation is significant at the 0.01 level.

Following the suggestion of one the reviews based on the inhibitory control account of the relationship between the non-symbolic comparison task and exact calculation [52], [53], we ran separate correlations between the *w* calculated from two sets of stimuli (*wSize*: size control; *wArea*: area control; see Methods) used in the non-symbolic comparison task and exact calculation. Partial correlations controlling for the effects of age, intelligence and spelling revealed that both *wSize* and *wArea* significantly correlated with exact calculation (*r* = −0.13, *p* = 0.033 and r = −0.175, *p* = 0.007, respectively). Importantly, Fisher's r-to-z transformation revealed that there was no significant difference between the two correlation coefficients (*z* = −0.459, *p* = 0.359). Therefore, we used the *w* calculated from all trials in further analyses.

Next, the specific contributions of the different instantiations of the ANS in exact calculation were determined by calculating three multiple regression models. In all three models, two blocks of variables were defined. In the first block, the intervening variables age, schooling, general intelligence and spelling abilities were added using the method “*enter*”. In the second block, the three measures of ANS acuity were included as single predictors in three separate models. The first block of variables explained 57.8% of the variance in exact calculation (see the coefficients in Table 4). Importantly, all three measures of ANS acuity remained significant predictors of exact calculation after removing the effects of the intervening variables (non-symbolic comparison: *β* = −0.135, *p* = 0.005; non-symbolic estimation: *β* = −0.162, *p*<0.001; non-symbolic addition: *β* = −0.167; *p*<0.001). This finding indicates that all three instantiations of the ANS contribute to explaining exact calculation independently of age, schooling, general intelligence and spelling abilities.

**Table 4:** Stepwise regression with exact calculation as the dependent variable and non-symbolic comparison, non-symbolic estimation and non-symbolic addition as predictors, regressing out the effects of age, schooling, general intelligence and spelling abilities.

Model	Predictors	*B*	*SE*	*Beta*	*t*	*p-value*
Block 1 *R^2^* = 0.578	Age	0.349	0.156	0.236	2.231	0.027
	Grade	8.736	1.862	0.485	4.692	<0.001
	Raven	8.882	1.530	0.302	5.805	<0.001
	TDE Spelling	8.330	1.719	0.237	4.845	<0.001
Block 2 *R^2^* = 0.037	Nsymb Addition (cv)	−34.688	12.689	−0.136	−2.734	0.007
	Nsymb Estimation (cv)	−29.214	11.568	−0.128	−2.525	0.012

TA  =  typically achieving; MD  =  mathematical difficulties; w: internal Weber fraction; cv: coefficient of variation.

Because all measures of ANS acuity are intercorrelated, the extent to which different instantiations of the ANS present unique contributions to exact calculation was determined. A multiple regression model was calculated with the same structure as before. The first block of variables included the same variables as before, and the second block of variables considered the three measures of ANS acuity simultaneously by using the “*stepwise*” method. The regression model kept non-symbolic estimation and non-symbolic addition but excluded non-symbolic comparison. Non-symbolic addition raised the variance explained to 60.1% and non-symbolic estimation to 61.4% (Table 4). Therefore, the multiple regression analysis showed that both non-symbolic estimation and non-symbolic addition have unique contributions to exact calculation. Moreover, the contribution of non-symbolic comparison to explain exact calculation is shared with other instantiations of the ANS.

Together, these results reveal that all of the instantiations of the ANS contribute to explaining exact calculation, but their contributions are not always unique. More specifically, the effects of non-symbolic comparison on exact calculation appear to be fully shared by non-symbolic estimation and non-symbolic addition. In contrast, a portion of the impact of these two variables on exact calculation appears to be unique. That is, the effect of non-symbolic comparison on exact calculation is indirect, because it is common to non-symbolic estimation and non-symbolic addition. Are these results due to mediation processes that act inside the ANS? To specifically test this hypothesis, one must test whether the effect of one instantiation of the ANS (X) on exact calculation (Y) is significantly absorbed by another instantiation of the ANS (M) [54], [55]. Moreover, to increase the confidence in the direction of the mediation effect, it is necessary to determine whether the effect of M in exact calculation is also reduced to the same extent by the inclusion of X as a mediator variable.

To investigate these possible mediation effects, we conducted Causal Mediation Analysis [54], [55], as implemented in the R package *mediation* (version 4.2.2)“ [56]. Six models that analyzed all of the possible combinations of different measures of ANS acuity as both predictors (X) and mediators (M) and exact calculation as the outcome (Y) were calculated. In each model, the total effect of each instantiation of the ANS on exact calculation was decomposed into a mediation and a direct effect. The regression coefficients as well as their confidence intervals and statistical significance are depicted in Table 5. To determine the statistical significance of the coefficient estimates, a nonparametric bootstrap method was employed. To obtain reliable estimates, a total of 10,000 samples for bootstrapping were drawn.

**Table 5:** Mediation models with measures of ANS acuity as either predictors (X) or mediators (M) and exact calculation as the outcome (Y).

Models	Variables	Effects	Estimate 95%	CI Lower 95%	CI Upper	*p-value*
1.1	X = Nsymb Comparison	Direct	−25.113	−43.782	−6.943	0.012
	M = Nsymb Estimation	Mediation	−7.665	−14.791	−1.864	0.038
1.2	X = Nsymb Estimation	Direct	−30.810	−50.139	−9.162	0.002
	M = Nsymb Comparison	Mediation	−6.239	−13.116	−1.337	0.069
2.1	X = Nsymb Comparison	Direct	−21.982	−45.660	−1.744	0.064
	M = Nsymb Addition	Mediation	−10.795	−19.664	−2.047	0.026
2.2	X = Nsymb Addition	Direct	−34.319	−60.102	−6.306	0.008
	M = Nsymb Comparison	Mediation	−8.308	−18.448	−0.791	0.094
3.1	X = Nsymb Estimation	Direct	−29.214	−48.779	−8.317	0.003
	M = Nsymb Addition	Mediation	−7.835	−15.859	−1.565	0.055
3.2	X = Nsymb Addition	Direct	−34.688	−59.325	−9.521	0.006
	M = Nsymb Estimation	Mediation	−7.939	−16.354	−1.630	0.058

X  =  predictor variable, M  =  mediator variable.

As can be seen in Table 5, only Models 1.1 (X  =  non-symbolic comparison, M  =  non-symbolic estimation) and 2.1 (X  =  non-symbolic comparison; M  =  non-symbolic addition) presented directional mediation effects (*p* = 0.038 and *p* = 0.026, respectively). These results revealed that both non-symbolic estimation and non-symbolic addition mediate the total effect of non-symbolic comparison on exact calculation. While non-symbolic estimation has a partial mediation effect, because the direct effect between non-symbolic comparison to exact calculation remained significant (*p* = 0.012), non-symbolic addition has a complete mediation effect, because the direct effect from non-symbolic comparison to exact calculation failed to reach statistical significance (*p* = 0.064). The direction of the possible causal direction between the measures of ANS acuity and exact calculation was further corroborated by the fact that the alternative models 1.2 and 2.2 with non-symbolic comparison as the mediator variable did not show any significant mediation effect (*p* = 0.069 and *p* = 0.094, respectively). Finally, the results revealed no mediation directional effects between non-symbolic estimation and non-symbolic addition to exact calculation (*p* = 0.055 and *p* = 0.058, respectively).

## Discussion

The present study investigated the relationship between three measures of ANS acuity (non-symbolic comparison, estimation and addition), their cross-sectional trajectories in children with typical and atypical arithmetic abilities, and their specific contributions to exact calculation. The children with MD were found to have impairments in multiple instantiations of the ANS, more specifically in non-symbolic comparison and non-symbolic addition. Moreover, the TA children were more accurate in mapping between non-symbolic magnitudes and number words compared to the children with MD, although this difference was only marginally significant.

Interestingly, the acuity of the non-symbolic comparison was found to develop normally in TA, but not in the MD group. The children with MD did not show any improvement with age in this task. A bootstrapping analysis confirmed that this difference was not due to a lack of statistical power given the smaller sample size of the MD group. Regarding the acuity of non-symbolic addition, both groups improved with age, but the children with MD were less accurate compared to their TA peers. Finally, both groups also improved to the same extent in the non-symbolic estimation task.

The three measures of ANS acuity significantly correlated with each other, which possibly reflects at least in part a common numerosity code, as proposed by Dehaene [34]. Importantly, all three measures of ANS acuity significantly correlated with exact calculation. However, a multiple regression analysis revealed that only non-symbolic estimation and addition contributed with unique proportions of variance in explaining exact calculation. Mediation analysis showed that the effect of non-symbolic comparison on exact calculation was mediated to different degrees by non-symbolic estimation and non-symbolic addition.

### Differences between the TA and MD groups in ANS acuity

In line with previous studies that have investigated the cognitive mechanisms that underlie MLD, the ANS acuity as measured by non-symbolic comparison was found to be impaired in children with MD compared to their TA peers [12], [13], [57]. Importantly, we were able to detect deficits in the ANS even in a group of MD children selected with a more liberal criterion, which probably includes children with high cognitive heterogeneity [43]. This finding lends support to the view that the different forms of MD are better described as a continuous spectrum rather than qualitatively different categories.

The children with MD were also found to be impaired in the acuity of non-symbolic addition. To our knowledge, this study was the first that demonstrated that non-symbolic addition is impaired in children with low achievement in math. De Smedt and Gilmore [38] found no impairment in a non-symbolic addition task in children with DD during the first year of formal schooling. However, the authors used a two-alternative forced choice task and analyzed only the mean accuracy of the responses. In the present study, the task used allowed children to compare their internally generated sum with five different options that were presented. Accordingly, the acuity of the internal representation of numbers could be determined by calculating the *cv*. Therefore, our measure is more sensitive to capturing differences.

Finally, in line with previous studies [12], [27], TA children were more accurate in mapping between non-symbolic stimuli and number words compared to children with MD, although in our study this difference was only marginally significant.

Both the MD and TA groups had normal intelligence (IQ>85) and normal spelling achievement (above the 25^th^ percentile), but group differences in those domains were still observed. For this reason, intelligence and spelling abilities were included as covariates in the group comparison analyses. These results are similar to other studies that also found medium to high effect sizes when comparing language-related abilities [13] and intelligence [58] between TA and DD groups. Lower language-related and intelligence performance could reflect the more widespread impairment that is frequently observed in children who have specific developmental disorders [59].

### Cross-sectional trajectories of ANS acuity

More detailed analyses of the cross-sectional trajectories of ANS acuity in typically and atypically developing children revealed several findings that merit discussion. First, while the *w* for the TA children decreased with age, this relationship was not the case for the children with MD. While longitudinal studies [16], [18] have found that ANS acuity measured by the non-symbolic comparison task prior to formal schooling is a specific predictor of later mathematics achievement in TA children, group studies with younger children (6 to 9 years old) failed to find differences in this task between TA and children with DD (for a review, see Noël & Rousselle [32]). However, studies with younger children did not use the *w* as an index of ANS acuity, which might be a more sensitive parameter to capture group differences compared to the commonly used distance effect (e.g. Oliveira-Ferreira et al. [45]). Furthermore, those studies tended to use a more liberal criterion to classify the children with DD, for example, below the 15^th^ percentile [35], [38]. In contrast, studies with older children that used a more stringent criterion to characterize the DD group (below the 10^th^/5^th^ percentile) reported an elevated *w* for these children compared to their TA peers [12], [13]. Because no correlation was found between non-symbolic comparison and age in children with MD as opposed to their TA peers, our results suggest that it might be easier to detect group differences in this task in older children, because differences seem to increase over the course of development.

Importantly, we were able to detect group differences in the non-symbolic comparison task in a group of 10-year-olds even when using a very liberal criterion to select children with math difficulty. The acuity of the non-symbolic addition was found to increase more or less to the same degree in both the TA and MD groups; however, TA children were systematically more accurate than the children with MD. This finding suggests that even from the initial years of formal schooling, MD children might already present a detectable impairment in more complex manipulations of numeric information. In line with the present results, non-symbolic addition measured before formal math instruction was found to be a specific predictor of later math achievement [15]. A similar pattern compared to non-symbolic addition was observed in the non-symbolic estimation task; however, a group comparison revealed that there was only a marginally significant result.

Taken together, the results suggest that the hypothesis put forward by Noël and Rousselle [32], which states that the ANS is not impaired in children with DD, based solely on the results of a single measure of ANS acuity (non-symbolic comparison) might be too simplistic. The present results indicate that compared to TA children, younger children with low achievement in math selected even with a liberal criterion already present a lower acuity in non-symbolic addition, which is a task that probably calls for more manipulations within the ANS than non-symbolic comparison. The characterization of cross-sectional trajectories can be considered as an important step towards the understanding of the evolution of developmental disorders, but should be confirmed in future longitudinal studies [60].

### Relationship between the measures of ANS acuity and exact calculation

As expected, significantly positive correlations were found between the three measures of ANS acuity. This finding is consistent with the data from Mazzocco et al. [12], who reported an association between non-symbolic comparison (*w*) and non-symbolic estimation (*cv*) in 14-year-old adolescents. However, the results are inconsistent with the only study that directly investigated the association between more than one measure of ANS acuity [41]. These authors found null correlations between the *w* calculated from non-symbolic versions of the number comparison and approximate addition tasks, suggesting that these tasks are measuring completely different constructs. However, this study has an important limitation, which is that for the non-symbolic comparison task, only three ratios were used to fit the psychometric function. As is known from the psychophysical literature, it is very difficult to have good fits from using only three points in the psychometric curve [61]. Thus, the lack of correlation reported by the authors could simply reflect a poor estimation of the coefficients. In contrast, in the present study, a much larger range of data points (eight) was used to calculate the *w* in the non-symbolic comparison task. Therefore, the moderate but significant correlations between non-symbolic comparison, non-symbolic estimation and non-symbolic addition found in the present study are compatible with the existence of different instantiations of the ANS which at least partially activate a common underlying numerosity code. Nevertheless we agree with both Gilmore et al. [41] and Park and Brannon [62] that it is very misleading to select only one task involving non-symbolic numerical stimuli and present it as a valid index of a supposedly unique ANS. This is a very frequent practice in the numerical cognition literature that should be avoided in further studies.

The indices *w* and *cv* correspond to the degree of noise in the internal representation of numerosity and are mathematically equivalent, in the sense that they are on the same scale (for a comprehensive review on the mathematical basis of the internal Weber fraction, see Dehaene [1]; for an intuitive explanation about the relationship between the Weber fraction and the coefficient of variation, see Halberda [63]). Indeed, the values of the parameters in the three tasks have been revealed to be similar (see Table 2). However, one should not expect them to be equal, because the tasks that were used to extract them involve very different cognitive processes: simple discrimination, mapping from non-symbolic magnitudes to number words and more complex manipulations of magnitudes in the context of an arithmetic operation, for non-symbolic comparison, estimation and addition. Interestingly, the coefficient values increased from non-symbolic comparison to non-symbolic addition; thus, it is tempting to speculate that this result possibly reflects the summation of noise during the process of accumulation of evidence in more complex forms of magnitude manipulation. For example, the internally generated sum of two given numerosities is possibly noisier than the representation of the numerosities themselves, because during addition there is another source of noise, which arises from the operation itself. In fact, the two existent mathematical models of approximate calculation [37], [64] account for this additional source of variation, by including a scaling factor that corresponds to the amount of noise due to the calculation. The same logic could be applied to non-symbolic estimation. In this task, one needs to not only discriminate the magnitudes but also transform the represented value in a symbolic label, which certainly corresponds to another source of noise [1]. The precise mechanisms that are involved in different instantiations of the ANS and how their interaction occurs is a very exciting topic, but scarcely addressed in the literature. Therefore, future studies should investigate empirically the different sources of noise during magnitude manipulations and their spatial-temporal neural dynamics.

Next, we investigated more deeply the association between measures of ANS acuity and exact calculation. Significant correlations between all three measures of ANS acuity and exact calculation were found. Importantly, all three measures of ANS acuity have specific contributions to explain variance in exact calculation, which remain significant after partialling out the more general effects of age, schooling, general intelligence and spelling abilities. Accordingly, the link between the different instantiations of the ANS and exact calculation does not seem to be generated by general cognitive processes, but rather by magnitude processing abilities underlying the tasks. However, our results are not definitive. It is still possible that other general cognitive abilities that we didn't include in this study, such as executive functions and especially inhibitory control [52], [53] can account for this link.

Previous studies also found that non-symbolic estimation [12], [27], [36] and non-symbolic addition [15] are significantly correlated with exact calculation. Regarding the non-symbolic comparison, the literature is more inconsistent and contains both positive and negative results (see De Smedt et al. [24] for a comprehensive review). As noted by De Smedt et al. [24], this inconsistency can probably be attributed to methodological differences in the non-symbolic comparison tasks, in the index calculated from behavior (e.g., RT, accuracy, distance effect, Weber fraction) and also in the math tests used. This inconsistency has led some authors to radically argue that the ANS does not make any contribution to explaining exact calculation [32]. However, this conclusion may have been premature, since two recent meta-analyses reported an association, although moderate, between non-symbolic comparison and math achievement from childhood to adulthood [25], [26]. Moreover, the present study also showed that partial correlations between ANS acuity and exact calculation are in the same order of magnitude as that reported by Chen and Li [26], and Fazio et al. [25], which cannot be reduced to more general cognitive processes. After inspecting all these results, one can be confident to assume the existence of a specific link between very basic ANS related processes and exact calculation.

An alternative hypothesis is that the non-symbolic comparison task is not a measure of the precision of numerical representations, but rather a measure of inhibitory control, since it is necessary to inhibit the processing of continuous visual parameters to be able to accurately discriminate between two numerosities [52], [53]. To ensure that participants are not using non-numerical variables to judge which collection of dots is the larger, researchers normally use different sets of stimuli varying continuous visual properties, such as dot size and dot total area. For example, Fuchs and McNeil [52] used three different sets of stimuli: dot total area was constant and dot size positively covaried with numerosity; mean dot size was constant and dot total area positively covaried with numerosity; dot total area and mean dot size were both inversely covaried with numerosity (‘inverse ANS acuity’ trials). Interestingly, results demonstrated that only the accuracy in the inverse ANS acuity set was significantly correlated with math achievement. Furthermore, accuracy in this set showed the highest correlation coefficient with a measure of inhibitory control. Similarly, Gilmore et al. [53] used two sets of stimuli: dot total area and dot size positively covaried with numerosity (‘congruent’ trials); dot total area and dot size negativelly covaried with numerosity (‘incongruent’ trials). Consistently with Fuchs and McNeil [52], results showed that incongruent trials were significantly correlated with math achievement but congruent trials were not.

As described in the Methods, in the present study we used two different sets of stimuli: dot size was constant and dot total area positively covaried with numerosity (size control); dot total area was constant and dot size negatively covaried with numerosity (area control). Therefore, we didn't have the ‘inverse ANS acuity’ [52] or the ‘incongruent’ [53] sets of trials. Nevertheless, we calculated the *w* separately for the size control and area control items. Partial correlations controlling for the effects of age, intelligence and spelling revealed that both *wSize* and *wArea* significantly correlated with exact calculation and no significant difference were found between the two coefficients. However, we cannot rule out the possibility that inhibitory skills could account at least in part for the relationship between the non-symbolic comparison task and exact calculation in the present study. It is important to note that a serious limitation of separating the items in two different categories, which the above-mentioned studies didn't take into account, is that the number of observations in each category of stimuli dramatically decreases and consequently compromises the stability of the measure. Therefore, we decided to use the *w* calculated from all items in all the analyses.

Importantly for our results, higher correlations between non-symbolic comparison and math abilities were reported in studies that measured math ability with standardized achievement batteries, which normally include items closely associated with the representation and manipulation of numerical quantity without invoking knowledge of arithmetic (e.g. TEMA-3 [11], [18]). In fact, more recently, Libertus, Feigenson and Halberta [65] analyzed the association between the non-symbolic comparison task and the items present in the widely used TEMA-3 separated in two distinct categories: items associated with informal (e.g. enumeration and number comparison) and formal (e.g. transcoding and exact calculation) mathematical abilities. Results demonstrated that the performance in the non-symbolic comparison task was only significantly correlated with informal mathematical abilities. Similarly, Piazza et al. [13] found the performance in the non-symbolic comparison task significantly correlated only with a subtest of a standardized math achievement battery that required children to compute the proximity relations between different numbers, but not with the other subtests, which measured transcoding and exact calculation abilities. Therefore, it is most likely that non-symbolic comparison, a very basic form of number manipulation within the ANS, has an indirect and consequently moderate effect in exact calculations.

Accordingly, Lyons and Beilock [39] found that the ability to order a series of digits fully mediated the correlation between non-symbolic comparison and basic symbolic arithmetical operations in adults. In the same line, van Marle et al. [40] found that the association between non-symbolic comparison and math achievement in children was also fully mediated by a series of symbolic numerical tasks, mainly the knowledge of cardinal value. Following the same logic, it is also possible that other instantiations of the ANS that require different forms of numerical manipulation also account for the effect of non-symbolic comparison in exact calculation. Indeed, results of our multiple regression model revealed that non-symbolic comparison did not uniquely contribute to explain the variance of exact calculation, when all three measures of ANS acuity were considered simultaneously. To our knowledge, no previous study has systematically investigated the effects of non-symbolic comparison, estimation and addition on exact calculation. In order to do that, we calculated six mediation models with all combinations of measures of ANS acuity as either predictors or mediators and exact calculation as the outcome. The results revealed first that non-symbolic estimation partially mediates the relation between non-symbolic comparison and exact calculation. This finding is in line with Mazzocco et al. [12], who demonstrated that both non-symbolic comparison and non-symbolic estimation accounted for unique proportions of variance in a math achievement task. Second, a full mediation effect of non-symbolic addition was found to be present in the relation between non-symbolic comparison and exact calculation. These results are fully compatible with the ones recently reported by in Park and Brannon [61], who demonstrated that the training on non-symbolic addition but not in non-symbolic comparison has a significant transfer effect to exact calculation. Therefore, the authors suggested that the active process of manipulating numerical information is the critical mechanism underlying the association between the basic number processing and exact calculation.

Although significantly correlated, the three ANS related tasks investigated in the present study involve different cognitive processes. The non-symbolic comparison task involves a very basic operation of magnitude discrimination, which is found to be already present in infancy. Differently, the non-symbolic estimation task involves a transcoding process from approximate to exact symbolic representations of numbers. Finally, the non-symbolic addition involves a more complex process of arithmetical transformations. Results of the mediation analyses show the existence of multiple associations between the different measures of ANS acuity and exact calculation and suggest the existence of a hierarchy of complexity between different instantiations of the ANS. These different instantiations seems to be organized from the more basic and less cognitively demanding forms of number processing to more elaborate operations that involve more active manipulation of magnitudes. The crucial evidence supporting this hypothesis is that alternative models with non-symbolic comparison as the mediator variable for the association of non-symbolic estimation and non-symbolic addition with exact calculation showed no significant mediation effects. At the neural level, this hierarchical organization of different processes underlying number representation and manipulation might reflect the increasing functional connectivity between and within the left and right parietal cortices, as observed during the performance of number-related tasks with increasing demands on the processing of numerical information [42]. Finally, the hierarchical structure of the different instantiations of the ANS can account for the finding that exact calculation is more strongly associated with non-symbolic estimation and non-symbolic addition, compared to non-symbolic comparison.

## Conclusions

Benefiting from high statistical power, we showed that children with MD, even when selected with a more liberal criterion, present lower acuity in multiple instantiations of the ANS (non-symbolic comparison and addition), even after controlling for the effects of intelligence and spelling abilities. This finding lends support to the view that the different forms of MD are better described as a continuous spectrum rather than qualitatively different categories. Second, the analyses of the cross-sectional trajectories showed that the ANS acuity measured by all three tasks positively correlated with age in TA children, while no correlation was found between non-symbolic comparison and age in the MD group. A plausible explanation for this result is that number discrimination, as the most basic form of numerical manipulation, is less prone to compensatory strategies that MD children could have developed to solve the other number-related tasks. Third, for the first time, we demonstrated that the three instantiations of the ANS investigated were significantly correlated among each other, reflecting at least in part a common numerosity code. Finally, mediation models revealed that non-symbolic estimation partially and non-symbolic addition fully mediated the effects of non-symbolic comparison in exact calculation. Therefore, the present study represents an important step towards a deeper understanding of the cognitive mechanisms underlying the relationship between basic number processing and mathematics. Given the highly hierarchical nature of mathematics, further studies should focus on precisely investigating the association between each instantiation of the ANS and different forms of mathematical reasoning. This will certainly help to a better understanding of the typical normal development of mathematical abilities as well as the nature of developmental dyscalculia.

## Supporting Information

Table S1 **Descriptive data of the individual assessment sample by grade.**
(DOCX)

Data S1 **Raw data.** Data_S1.zip. (sheet 1: data; sheet 2: variables specifications).
(ZIP)
