# Supplementary material for: In How Many Ways is the Approximate Number System Associated with Exact Calculation?
Source: PLoS One. 2014 Nov 19;9(11):e111155. doi: 10.1371/journal.pone.0111155 (PMC4237330; doi:10.1371/journal.pone.0111155)
Supplement: Table S1 — Descriptive data of the individual assessment sample by grade. (DOCX) [file pone.0111155.s001.docx]

**Supporting Information**

**Tables**

Table S1: Descriptive data of the individual assessment sample by grade.

**Dataset**

**Raw data**

Data_S1.zip

(sheet 1: data; sheet 2: variables specifications)

Table S1: Descriptive data of the individual assessment sample by grade.

| Variables | 1th and 2th grade | | | | 3th and 4th grade | | | | 5th and 6th grade | | | |
| --- | --- | --- | --- | --- | --- | --- | --- | --- | --- | --- | --- | --- |
|  | TA (n = 31) | | MD (n = 9) | | TA (n = 105) | | MD (n = 27) | | TA (n = 26) | | MD (n = 4) | |
|  | Mean | SD | Mean | SD | Mean | SD | Mean | SD | Mean | SD | Mean | SD |
| Age (months) | 104.10 | 4.47 | 97.00 | 7.73 | 121.45 | 7.37 | 121.96 | 11.14 | 142.85 | 9.56 | 141.75 | 9.74 |
| Raven (IQ) | 116.57 | 10.21 | 100.80 | 7.23 | 110.40 | 10.26 | 105.97 | 9.90 | 104.35 | 8.27 | 94.11 | 5.54 |
| TDE Arithmetics | 114.40 | 11.40 | 85.73 | 4.80 | 107.05 | 10.80 | 86.07 | 5.40 | 109.90 | 12.30 | 83.95 | 3.98 |
| TDE Spelling | 112.79 | 9.11 | 92.09 | 9.98 | 109.30 | 7.90 | 103.38 | 6.60 | 109.90 | 7.35 | 107.20 | 6.90 |

*TA: typically achieving; MD: mathematical difficulties. Both TDE Arithmetics and TDE Spelling scores are in a standardized form with mean = 100 and SD = 15.*
